# Quantitative assessment of interstitial lung disease in Sjögren’s syndrome

**DOI:** 10.1371/journal.pone.0224772

**Published:** 2019-11-08

**Authors:** Pablo Guisado-Vasco, Mario Silva, Miguel Angel Duarte-Millán, Gianluca Sambataro, Chiara Bertolazzi, Mauro Pavone, Isabel Martín-Garrido, Oriol Martín-Segarra, José Manuel Luque-Pinilla, Daniele Santilli, Domenico Sambataro, Sebastiano E. Torrisi, Ada Vancheri, Marwin Gutiérrez, Mayra Mejia, Stefano Palmucci, Flavio Mozzani, Jorge Rojas-Serrano, Carlo Vanchieri, Nicola Sverzellati, Alarico Ariani

**Affiliations:** 1 Internal Medicine, Complejo hospitalario Ruber Juan Bravo, Universidad Europea (Madrid), Madrid, Spain; 2 Scienze Radiologiche, Dipartimento di Medicina e Chirurgia (DiMeC), University of Parma, Parma, Italy; 3 Internal Medicine, Hospital universitario Fuenlabrada, Fuenlabrada, Spain; 4 Regional Referral Center for Rare Lung Diseases, A. O. U. "Policlinico-Vittorio Emanuele" Dpt. of Clinical and Experimental Medicine, University of Catania, Catania, Italy; 5 Division of Musculoskeletal and Rheumatic Disorders, Instituto Nacional de Rehabilitación—“Luis Guillermo Ibarra Ibarra”, Mexico City, Mexico; 6 Internal Medicine and Rheumatoloy Unit, Azienda Ospedaliero-Universitaria di Parma, Parma, Italy; 7 Department of Clinical and Experimental Medicine, Internal Medicine Unit, Cannizaro Hospital, University of Catania, Catania, Italy; 8 Interstitial Lung Disease and Rheumatology Unit, Instituto Nacional de Enfermedades Respiratorias, Ismael Cosio Villegas, Mexico City, Mexico; 9 Department of Medica Surgical Sciences and Advanced Technologies "GR Ingrassia", Radiology I unit, University of Catania, Catania, Italy; Nippon Medical School, JAPAN

## Abstract

**Background:**

Interstitial lung disease (ILD) is a frequent manifestation of Sjögren’s syndrome (SS), an autoimmune disease of salivary and lacrimal glands, and affects approximately 20% of patients. No clinical or serological features appear to be useful to predict its presence, severity or progression, and chest high-resolution computed tomography (CT) remains the gold standard for diagnosis. Semiquantitative CT (SQCT) based on visual assessment (Goh and Taouli scoring) can estimate ILD extent, although it is burdened by relevant intra- and interobserver variability. Quantitative chest CT (QCT) is a promising alternative modality to assess ILD severity.

**Aim:**

To determine whether QCT assessment can identify extensive or limited lung disease in patients with SS and ILD.

**Methods:**

This multi-center, cross-sectional and retrospective study enrolled patients with SS and a chest CT scan. SQCT assessment was carried out in a blinded and centralized manner to calculate both Goh and Taouli scores. An operator-independent analysis of all CT scans with the open-source software platform Horos was used to evaluate the QCT indices. Patients were classified according to the extent of ILD and differences in QCT index distribution were investigated with non-parametric tests.

**Results:**

From a total of 102 consecutive patients with SS, the prevalence of ILD was 35.3% (36/102). There was a statistically significant difference in QCT index distribution between the SS with ILD and SS without ILD groups (p<0.001). Moreover, SS-ILD patients with ILD >20% (by Goh score) had a QCT index statistically different from those with limited ILD extent (p<0.001). Finally, QCT indices showed a moderate-to-good correlation with the Goh and Taouli scores (from 0.44 to 0.65; p<0.001).

**Conclusions:**

QCT indices can identify patients with SS and ILD and discriminate those with lesser or greater lung disease.

## Background

Sjögren’s syndrome (SS) is systemic autoimmune disease characterized by chronic lymphocytic inflammation of ductal epithelial structures. The disease affects principally the exocrine glands, in particular the lacrimal and salivary glands, causing dryness of mucosal surfaces–termed sicca syndrome–and glandular parenchymal damage. The pathogenesis of SS is poorly understood, and manifestations are heterogeneous [1]. Accordingly, there is increasing interest in its systemic involvement [2] and how to reach a precise diagnosis using molecular profiling [3–4].

Interstitial lung disease (ILD) remains one of most frequent pulmonary complications in primary SS and sub-clinical disease is even more common [5–6]. Pulmonary disease is a singular clinical and histopathological scenario among the organ-specific SS involvement and leads to increased risk of mortality [7–8]; and it is considered in the high systemic activity domain of the European League against Rheumatism (EULAR) Sjögren’s syndrome disease activity index (ESSDAI).

An accurate differential diagnosis of ILD, obstructive disease, bronchial hyper-responsiveness, bronchiolitis, bronchiectasis or xerotrachea can be challenging [9]. High-resolution chest computer tomography (HRCT) is currently considered the gold standard imaging modality in confirming a diagnosis of SS and has clear improvements over classical chest x-ray [10–12]. It also allows for the discrimination and estimation of the extent of the ILD and can inform on treatment decisions. Likewise, semiquantitative CT (SQCT) assessment, such as the Goh [13] and Tauli [14] visual scores, can estimate ILD severity–in terms of limited or extensive disease–in autoimmune systemic diseases. However, these classification systems suffer from intra- and interobserver variability [15]. Quantitative CT (QCT) analysis is a promising tool to assess primary or secondary ILD and its extent. It is based on software providing highly accurate and operator-independent measurements, termed QCT indices [16], which have been rapidly developed and validated in specific clinical settings, including systemic autoimmune diseases [17].

To the best of our knowledge, there have been no studies focused on the use of QCT for SS with ILD. Herein, we designed a study to evaluate whether QCT indices have clinical utility to screen for ILD and to appraise differences between limited and extensive ILD.

## Methods

We performed a multicenter, cross-sectional, and retrospective study in patients with SS enrolled in four university-affiliated centers.

The following inclusion criteria were applied: 2016 American College of Rheumatology (ACR)/EULAR criteria [18], a chest CT scan ordered by a primary care physician for any reason, and age older than 18 years. The exclusion criteria included those specified by the 2016 ACR/EULAR consensus, including IgG4 disease, any immunosuppressive therapy with any biological agent in the last 3 months, or methotrexate or leflunomide in the year prior to study inclusion. Prednisone (or equivalent) at a dose ≤7.5 mg (on a tapering plan only) or low-dose hydroxychloroquine was allowed. The research protocol was approved by the local ethics committees and was conducted in accordance with the tenets of the Helsinki Declaration. The protocol was developed following the STROBE statement [19]

The following data were collected from all patients: demographic variables (age, sex), date of disease onset, symptoms suspicion of pulmonary disease, smoking habit, chest CT scan, pulmonary function tests [diffusing capacity of carbon monoxide (DLco), DLco divided by alveolar volume (DLco/VA), forced vital capacity (FVC) and total volume capacity (TLC)], and autoantibodies profile (SSA/Ro, SSB/La). The laboratories of all the participating centers used the same methodology, which was adopted from the current standards of the American Thoracic Society/European Respiratory Society.

A DICOM (digital imaging and communications in medicine) viewer, open-source software (Horos **www.horosproject.org**) was used for analyses and the following QCT indices were obtained after scan processing: kurtosis (Kurt), mean lung attenuation (MLA), skewness (Skew) and standard deviation (Sdev). The entire procedure including the lung segmentation algorithm was performed as described previously [16]. Accordingly, the region of interest between -950 HU and 400 HU was considered as ‘pulmonary parenchyma’ (namely, lung parenchyma without vessels or bronchioles and not affected by fibrosis). Those voxels included in the whole lung volume with higher HU values were identified as non-parenchymal structures. The QTC indices were calculated according to these definitions, as parenchymal (i.e., pKurt, pSkew, etc.) and total (i.e., tKurt, tSkew, etc.) QTC. These indices are based on the histograms obtained from the computed analysis of the volumetric region of interest [17, 20].

All CT images were centrally and blindly reviewed by the same board-certified radiologists (MS, NS) and QCT indices were then calculated. The SQCT assessment was carried out to calculate both Goh and Taouli scores for each CT scan. On the basis of the SQCT assessment, two groups were established: patients with SS and with/without ILD.

Data were reported as mean and SD for continuous variables and numbers and percentages for categorical variables. The Kolgomorov-Smirnov test was used to check the assumption of normality of the continuous variables. Differences between subgroups were analyzed using a non-parametric test, as appropriate. The Spearman rank test was used to determine correlations of QCT indices with SQCT scores, pulmonary function tests, and the other collected variables. A Youden index, according to the area under the curve, was run to select the best cut-off point value of QCT indices to assess the ILD.

The research was approved by the ethics committee of Fundación Jiménez Díaz, Madrid.

Approval Number: PIC 17–2017 The data were analyzed anonymously.

A two-tailed probability of p<0.05 was considered statistically significant. All analyses were conducted using SPSS 20.0 (Chicago, IL, USA) and R (version 3.5.2) statistical software package (**http://www.R-project.org/**) (Vienna, Austria).

## Results

A total of 102 patients were enrolled between January 2017 and September 2018. Patient characteristics are listed in Table 1.

**Table 1:** Baseline characteristics of the patients with Sjögren’s syndrome.

	Total cohort	SS without ILD	SS-ILD	p-value
N	102	66	36	-
Age, median (yrs) (95% CI)	69 (65–71)	68 (63–71)	69 (63–74)	nss
Sex (M:F)	7:95	3:63	4:32	nss
Smoker (no:former:yes)*	74:8:11	47:8:4	27:0:7	nss
Disease duration, median (yrs) (95% CI)	4 (3–5)	3 (2–5)	5 (3–7)	nss
pSS prevalence (%)	79	83	72	nss
Onset symptoms (sicca:dyspnoea:other)	51:27:24	41:7:18	10:20:6	<0.001
Antibodies Ro/SSA prevalence (%)	52	47	63	nss
Antibodies La/SSB prevalence (%)	25	21	31	nss
FVC (%) (95% CI) **	97 (93–108)	108 (93–116)	94 (83–99)	0.03
DLco (%) (95% CI) ***	71 (62–81)	82 (73–87)	63 (53–71)	0.01
ILD pattern (NSIP:UIP:Other)	-	-	27:6:3	-
Goh score (95% CI)	0	0	12,5 (7.7–25.3)	-
Taouli score (95% CI)	0	0	8,0 (5.7–11.0)	-

Abbreviations: M, male; F, female; CI, confidence interval; pSS, primary SS; ILD, interstitial lung disease; Nss, not statistically significant; FVC, forced vital capacity; DLCO, diffusion of lung CO; TLC, total lung capacity; FEV1, forced expiratory volume in first second; NSIP, non-specific interstitial pneumonia; UIP, usual interstitial pneumonia.

* 9/102 patients data missing.

** 41/102 patients data missing.

*** 58/102 patients data missing

The diseases associated to the development of SS, secondary SS, were: rheumatoid arthritis (5,9%), systemic lupus erythematosus (2%), systemic sclerosis (12,7%), and undifferentiated connective tissue disease (9,8%). Of the, just 10 cases have associated ILD.

Pulmonary function tests were incomplete in more than half of all patients (41% and 58% of patients did not have FCV and DLco data, respectively). No differences were found for patients with SS with ILD (SS-ILD) and those without ILD in terms of age, disease duration and autoimmune profile. The most common onset symptom in the SS-ILD group was dyspnea (52%), whereas mouth or eye dryness was the most common onset symptom in the SS without ILD group (59%). Pulmonary function tests showed that %FCV and %DLco were lower in the SS-ILD group than in the SS without ILD group (p = 0.03 and p = 0.01, respectively). As expected, there was a strong correlation between the Goh and Taouli scores (rho = 0.98; p<0.001). Table 2.

**Table 2:** Correlations of quantitative indices and semiquantiative methods and lung function tests.

	*pKurt*	*pMLA*	*pSdev*	*pSkew*	*tKurt*	*tMLA*	*tSdev*	*tSkew*
*Goh score* *N = 102*	-0,58	0.48	0.55	-0,56	-0,62	0.44	0.36	-0,65
*Taouli score* *N = 102*	-0.58	0.48	0.56	-0.55	-0.63	0.45	0.36	-0.65
*PTF FVC (%)* *N = 61*	0.52	-0.55	-0.48	0.52	0.49	-0.41	-0.33 p = 0.03	0.49
*PRF DLco* *N = 44*	0.61	-0.54	-0.58	0.6	0.39 p = 0.009	-0.51	-0.22 ns	0.46 p = 0.002
*PFT TLC* *N = 27*	0.77	-0.64	-0.71	0.76	0.63	-0.49	-0.34 ns	0.70
*PFT FEV1 (%)* *N = 61*	0.4 p = 0.002	-0.43	-0.34 p = 0.007	0.39 p = 0.002	0.48	-0.41 p = 0.001	-0.29 p = 0.03	0.49

All correlations have a p-value <0.001, except where specified. Abbreviations. ILD, interstitial lung disease; p(X), pulmonary quantitative indices, t(X), total quantitative indices. Kurtosis (Kurt), Sweetness (Skew), standard deviation (Sdev) and mean lung attenuation (MLA). PFT, pulmonary function test; FVC, forced vital capacity; DLCO, diffusion of lung carbon monoxide; TLC, total lung capacity; FEV1, forced expiratory volume in first second.

All QCT indices (with the exception of tSdev) had a good correlation with the Goh and Taouli scores (rho ranges from 0.36 to 0.65; p<0.001). The QCT indices (except for tSdev) strength of correlation with FVC and DLco ranged from moderate to good (rho from 0.33 to 0.55 and from 0.39 to 0.61, respectively; p<0.001). Data are reported in Table 1.

Both Goh and Taouli scores had a moderate strength of correlation with FVC (rho = -0.36 and -0.38, respectively; p<0.004) and with DLco (rho = -0.42 and -0.44, respectively; p<0.004).

In the SS-ILD group, 44% (16/36) of patients had extensive lung disease (Goh score ≥20%). These patients had similar characteristics to those with limited SS-ILD (Table 3), except for lower FVC and DLco values, and a lower prevalence of nonspecific interstitial pneumonia (69% *vs* 80%).

**Table 3:** Characteristics of SS-ILD patients with limited *versus* extensive lung disease.

	SS-ILD cohort	SS-ILD (limited)	SS-ILD (extensive)	p
N	36	20	16	-
Age, median (yrs) (95% CI)	69 (63–74)	68 (62–76)	70 (61–75)	nss
Sex (M:F)	4:32	2:18	2:14	nss
Smoke habit (no:former:yes) *	27:0:7	16:0:3	11:0:4	nss
Disease duration, median (yrs) (95% CI)	5 (3–7)	6 (3–12)	4 (1–7)	nss
pSS prevalence (%)	72	80	63	nss
Onset symptoms (sicca:dyspnoea:other)	10:20:6	5:11:4	5:9:2	nss
SSA prevalence (%)	63	70	56	nss
SSB prevalence (%)	31	35	25	nss
FVC (%)(95% CI) **	94 (83–99)	100 (86–115)	84 (73–97)	0.03
DLCO (%)(95% CI) ***	63 (53–71)	70 (61–85)	51 (47–65)	0.02
ILD pattern (NSIP:UIP:Other)	27:6:3	16.1:3	11:5:0	0.05
Goh score (95% CI)	12,5 (7.7–25.3)	7.0 (4.0–8.0)	28.5 (25.6–46.7)	-
Taouli score (95% CI)	8,0 (5.7–11.0)	5.0 (2.2–6.0)	13.0 (11.0–13.7)	<0.001

Abbreviations: M, male; F, female; CI, confidence interval; pSS, primary SS; ILD, interstitial lung disease; Nss, not statistically significant; FVC, forced vital capacity; DLCO, diffusion of lung CO; TLC, total lung capacity; NSIP, non-specific interstitial pneumonia; UIP, usual interstitial pneumonia.

* 2/36 missing data

** 7/36 missing data

*** 13/36 missing data

**Fig 1:**
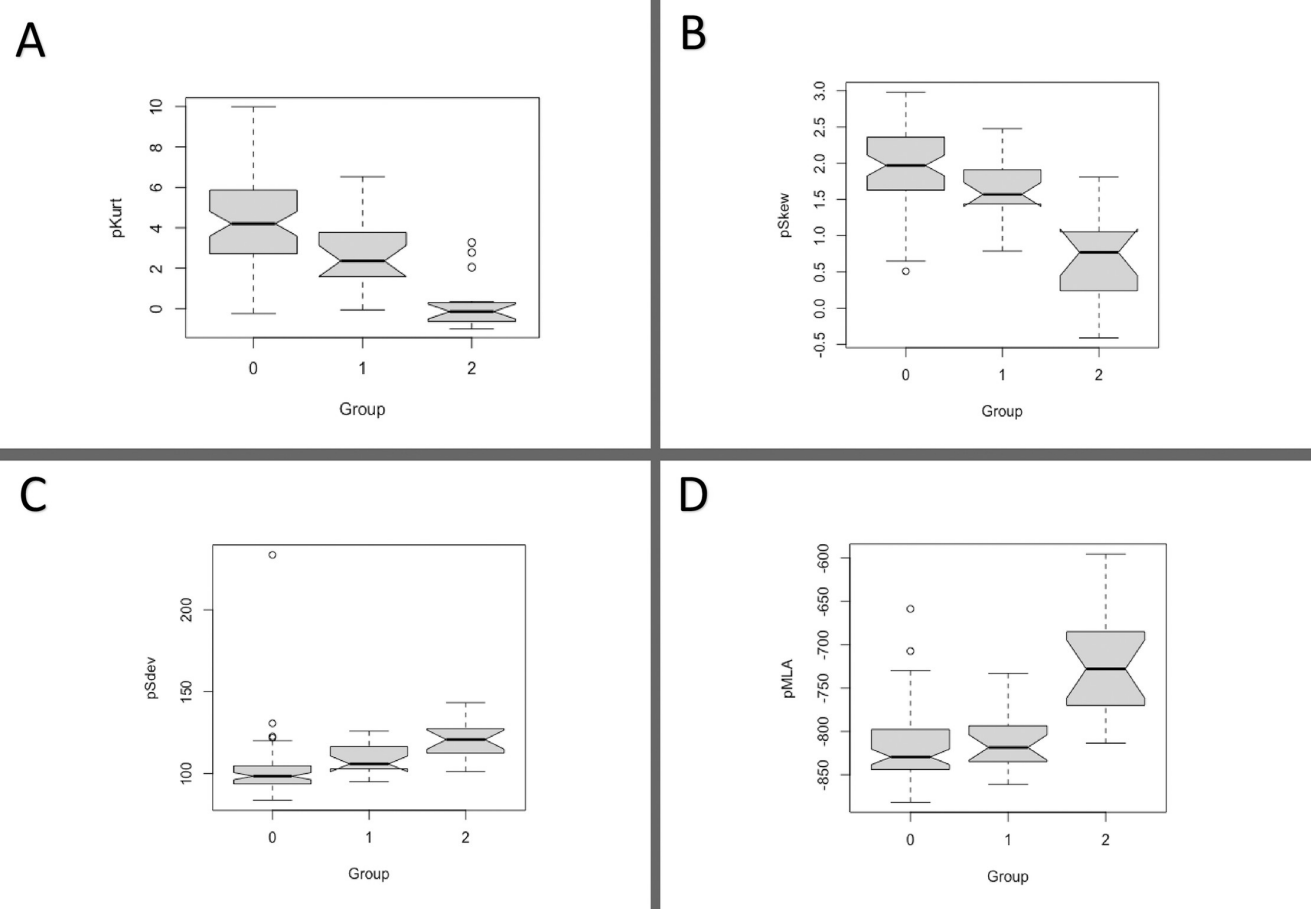
Quantitative CT indices distribution in Sjögren’s syndrome according to non-affected (group 0), limited ILD (group 1) and extensive (group 2) ILD. A. Pulmonary kurtosis; B. Pulmonary skewness; C. Pulmonary standard deviation; D. Pulmonary mean lung attenuation.Differences through multiple comparisons. A. Group 0 vs 1, p = 0.011; group 1 vs 2, p = 0.003; group 0 vs 2, p< 0.001. B. Group 0 vs 1, p = 0.07; group 1 vs 2, p<0.001; group 0 vs 2, p< 0.001.C. Group 0 vs 1, p = 0.28; group 1 vs 2, p = 0.12; group 0 vs 2, p< 0.001.D. Group 0 vs 1, p = NS; group 1 vs 2, p<0.001; group 0 vs 2, p< 0.001.

**Fig 2:**
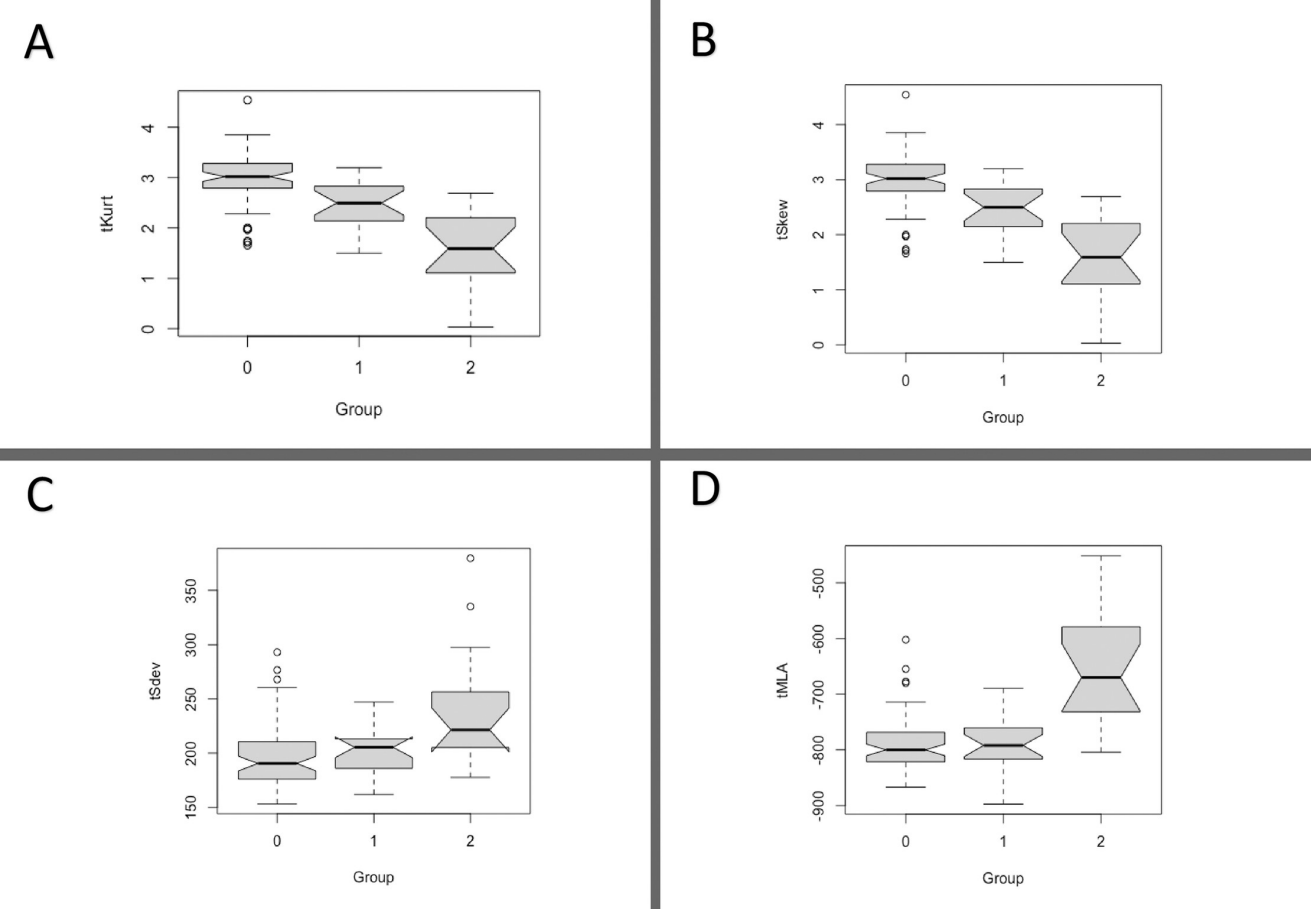
Quantitative CT indices distribution in Sjögren’s syndrome according to non-affected (group 0), limited ILD (group 1) and extensive (group 2) ILD. A. Total kurtosis; B. total skewness; C. Total standard deviation; D. Total mean lung attenuation. Differences through multiple comparisons. A. Group 0 vs 1, p = NS; group 1 vs 2, p = 0.04; group 0 vs 2, p = 0.03. B. Group 0 vs 1, p = 0.001; group 1 vs 2, p<0.001; group 0 vs 2, p< 0.001.C. Group 0 vs 1, p = NS; group 1 vs 2, p = 0.004; group 0 vs 2, p< 0.001.D. Group 0 vs 1, p = NS; group 1 vs 2, p<0.001; group 0 vs 2, p< 0.001.

All QCT indices except tSDev had a different distribution in the SS-ILD *versus* SS without ILD (p<0.001) group–defining the groups as follows: 0, SS non-affected; 1, SS limited ILD; and 2, SS extensive ILD. After clustering the SS-ILD patients according to ILD extent, the QCT indices (except for tSDev) had a statistically different distribution in the three subgroups (Fig 1 and Fig 2).

Of all QCT indices, tSkew and tKurt were the best ones to differentiate ILD pattern, or not, according to AUC, 0.87 (CI95% 0.79–0.94) and 0.84 (CI95% 0.76–0.93), respectively (Table 4).

**Table 4:** Cut-off point of quantitative indices according to the Youden index, and its corresponding sensitivity and specificity, to diagnosis interstitial lung disease in Sjögren’s syndrome.

	*Cut-off point*	*AUC*	*CI95%*	*Sensitivity (CI95%)*	*Specificity* *(CI95%)*	*Best p value*
pKurt	2.97	0.81	0.73–0.9	0.73(0.6–0.83)	0.81(0.64–0.92)	<0.001*
^Δ^pMLA	-826.2	0.74	0.64–0.84	0.83(0.67–0.94)	0.58(0.45–0.7)	<0.001
^Δ^pSdev	104.5	0.82	0.73–0.9	0.78 (0.61–0.9)	0.71(0.59–0.82)	<0.001
pSkew	1.66	0.8	0.71–0.89	0.74(0.62–0.84)	0.78(0.61–0.9)	<0.001*
pVol	3318.7	0.67	0.55–0.79	0.71(0.59–0.82)	0.64(0.46–0.79)	0.004
tKurt	7.62	0.84	0.76–0.93	0.85(0.74–0.92)	0.78(0.61–0.9)	<0.001*
tMLA^Δ^	-773.0	0.71	0.6–0.82	0.64(0.46–0.79)	0.73(0.6–0.83)	<0.001
tSdev^Δ^	200.6	0.69	0.58–0.79	0.72(0.55–0.86)	0.65(0.52–0.76)	0.002
tSkew	2.72	0.87	0.79–0.94	0.82(0.7–0.9)	0.81(0.64–0.92)	<0.001*
tVol	2937.08	0.62	0.5–0.75	0.92(0.83–0.97)	0.42(0.26–0.59)	0.04

Abbreviations. ILD, interstitial lung disease; p(X), pulmonary quantitative indices, t(X), total quantitative indices. Kurtosis (Kurt), Sweetness (Skew), standard deviation (Sdev), volume (Vol) and mean lung attenuation (MLA).

* Both pKurt/pSkew and tKurt/tSkew, were statistically equivalent. ^*Δ*^ Assuming normal pulmonary patterns.

## Discussion

To the best of our knowledge, this is the first study showing that QCT indices can characterize subjects with SS -ILD as compared to the standard visual semi-quantitative methods.

Pulmonary manifestations in SS (e.g., asthenia, cough, dyspnea) are variable in intensity and severity, and are often present before a diagnosis of SS is made. The prevalence of lung involvement in SS reported in different series ranges from 12 to 61%, which underscores the clinical necessity of a correct diagnosis [21]. Moreover, abnormalities in pulmonary parenchyma can be found in up to 50% of cases and an abnormal pulmonary function test typically reflects a restrictive (lung) rather than an obstructive (airways) pattern [9]. Whereas a reduction in DLco is generally the most common abnormality, pulmonary function tests are frequently unable to correctly describe lung involvement in SS–being more accurate in the advanced stage of the disease–and hence have poor sensitivity to detect subclinical pulmonary involvement. Accordingly, HRCT quantification could be useful for monitoring disease, its evolution and response to therapies. The QCT indices described here provide an operator-independent assessment of lung involvement by ILD, as compared with the Taouli and Goh scores, which are operator-dependent. Indeed, the latter score results might be ambiguous in a proportion of cases, even when combined with abnormalities in pulmonary function tests, and may be unable to correctly classify some cases in the corresponding category of severity. By contrast, QCT measurements have proven to provide highly accurate and reproductible diagnoses [15], although they require some level of training to follow a standardized imaging acquisition protocol. Moreover, fully automated QCT measurements could eliminate intra- and interobserver variability, particularly when used in diagnostic decision making.

The performance of QCT indices has been previously explored in other autoimmune diseases such as systemic sclerosis (SSc). For instance, in a recent series by Ariani et al., the authors reported a moderate-to-good agreement of all values for ILD associated with SSc, and also in cases with extensive or limited lung disease. Some pulmonary function tests also showed a relatively good correlation with QCT indices (FVC and DLco <70%) (16). Moreover, QTC indices could distinguish between high and low mortality groups [22] in those cases of SSc with ILD, in relation to 1-year mortality prediction clinical scales such as ILD-GAP score (ILD subtype, gender, age, FVC and DLco) or dBi (age, history of respiratory hospitalization, and FVC value and its change after 6 months).

Few studies have quantified pulmonary fibrosis related to SS. In the present study, we compared, for the first time, a QCT analysis of lung involvement against a specific SQCT score (Taouli) and a generic SQCT score (Goh) developed for secondary pulmonary fibrosis. The Goh score, which has been validated in SSc [13,23] and rheumatoid arthritis [24], here it shows excellent correlation with the more complex Taouli score. Hence, the Goh score appears a suitable method also for the quantification of SS-ILD. Likewise, it is reasonable to presume that the Goh score might have a similar predictive value for mortality [25, 26].

Although used as the only laboratory criteria, serologic/immunological parameters are not a definitive guide to diagnosis or to monitor the severity of ILD in SS, and the correlations across different studies are heterogenous. Indeed, we failed to find any significant association between QTC indices and anti-Ro, anti-La or ANA titers. However, anti-Ro/SSA titers were low in the general cohort but were higher in the ILD cohort.

The influence of the principal immunological markers on SS disease diagnosis was recently addressed by Brito-Zerón and colleagues using a Big Data analysis approach in 10,500 patients [27]. Regarding the phenotypes of patients, the frequency of the immunological markers ANA, Ro and La were quite similar in the pulmonary domain of the ESSDAI (approximately 10%) at diagnosis. When the authors analyzed the impact of three combinations of anti-Ro/La antibodies, no statistically differences were found in the pulmonary domain. These analyses suggest that novel autoantibodies should be developed to detect ILD with sufficient sensitivity or specificity. The use of more sophisticated profiling should be incorporated as soon as possible in the daily clinical practice, for example, the ratio of blood T cells [28], or microRNA profiles [29].

In a cross-sectional study aimed to evaluate the prevalence of respiratory symptoms in SS, Kampolis et al. found that up to 20% of all cases were affected [10]. As described in the aforementioned study, it is important to differentiate those respiratory symptoms/complaints that have an onset prior to SS diagnosis, such as any underlying respiratory disease–chronic obstructive pulmonary disease, bronchial asthma, or upper chronic airway cough–as approximately one-third of these cases had an established chronic respiratory disease that preceded the onset of SS. Along this line, the impact of smoking (or former smokers) is not infrequent; however, it should be differentiated from symptoms directly related to pulmonary involvement in SS. In some selected cases, however, both diseases could coexist in smokers with SS. Interestingly, the same authors did not report any specific alterations in pulmonary function tests (FEV_1_, FVC, ratio FEV/FVC, DLco) in those patients with pulmonary disease and SS. In our series, there were some missing data for smoking habit in a small percentage of patients (<10%). Thus, we believe that the issue concerning smoking habit does not significantly impact our results.

Theoretically, pulmonary function tests might not be substantially modified in ILD in SS until the disease is more advanced. Regarding this issue, lung ultrasound showed a good performance when compared with high-resolution thorax CT [30]. Indeed, this modality could be potentially useful to detect ILD earlier in SS, independently of the patient’s complaints; however, more research is needed to better understand the precise use of ultrasound as an imaging tool for identification of ILD and SS.

Our study has some limitations that should be considered, such as its retrospective design. Also, the CT protocols might be not homogenous across the participating centers–although the percentage of pulmonary disease in the global series fits well with recent data. Some differences could be expected in the immunological profile between ethnic groups and may affect the prevalence of ILD in our series [31]. Also, some degree of variable conditions when performing the CT images across the participating centers should be assumed–for example, a degree of heterogenicity in multiple slices and time points [32]. Another limitation is that while the reported pulmonary functional tests did not show obstructive profiles in most of the cases, FVC and DLco were not performed in all enrolled cases. Pulmonary function tests were often not requested at the same time as CT by the physicians. The reasons for this irregularity might be that they are not systematically assessed in SS in daily practice–considering they are likely not sufficient to help the clinician assess the extent of the ILD, or its severity. Some other biological/immunological profiles, such as cryoglobulinemia [8,27], were not collected. Our pooled analysis included primary and secondary SS, and this might impact on the QCT indices and patterns of ILD [33]. Finally, the QCT scores might be also influenced by the differences in ILD patterns in SS, such as bronchiolitis, bronchiectasis, non-specific interstitial pneumonia, usual interstitial pneumonia, lymphocytic interstitial pneumonitis, or organizing pneumonitis, among others.

QTC indices are becoming a useful tool in imaging analyses because they improve consistency of imaging diagnosis and might aid the treatment decisions in patients with ILD. This method is also promising for patient stratification according to ILD severity and extent [11, 22, 25, 26, 34]. In clinical practice, a quantitative ILD assessment with a user-friendly staging system (i.e., QCT index) could improve the outcomes of proposed SS-ILD treatments [35, 36].

Finally, the operator-independent algorithm we used in this study is free and time-saving. Accordingly, this method might be extremely suitable for multi-center trials focused on ILD. QCT indices are a promising alternative to visual scorings in ILD related to autoimmune diseases such as SS. We believe that this innovative tool will open new horizons for research into SS, as it has the capability to select ILD patients with extensive lung impairment and, correspondingly, a worse prognosis. QTC indices might potentially represent a pivotal tool at the time of diagnosis, and through management of ILD associated with SS.

## References

[1] Gao H, Zou YD, Zhang XW, He J, Zhang J, Sun Y et al Interstitial lung disease in non-sicca onset primary Sjögren’s syndrome: a large-scale case-control study. Int J Rheumatol Dis 2018; 21(7):1423–1429. 10.1111/1756-185X.13334 29968329

[2] Ramos-Casals M, Brito-Zerón P, Seror R, Bootsma H, Bowman SJ, Dörner T et al on behalf of, EULAR Sjögren Syndrome Task Force. Characterization of systemic disease in primary Sjögren’s syndrome: EULAR-SS Task Force recommendations for articular, cutaneous, pulmonary and renal involvements. Rheumatology (Oxford) 2017; 54(12):2230–2238. 10.1093/rheumatology/kev200 PMC6281074 26231345

[3] Delaleu N, Mydel P, Kwee I, Brun JG, Jonsson MV, Jonsson R. High fidelity between saliva proteomics and the biologic state of salivary glands defines biomarker signatures for primary Sjögren’s syndrome. Arthritis Rheumatol 2015; 67:1084–1095. 10.1002/art.39015 25545990

[4] Fischer A, Swigris JJ, du Rois RM, Groshong SD, Cool CD, Sahin H et al Minor salivary gland biopsy to detect primary Sjögren’s syndrome in patients with interstitial lung disease. Chest 2009; 136(49): 1072–1078. 19429722 10.1378/chest.08-2839 PMC3662204

[5] Ciancio N, Pavone M, Torrisi SE, Vancheri A, Sambataro D, Palmucci S, et al Contribution of pulmonary function tests (PFTs) to the diagnosis and follow up of connective tissue diseases. Multidisclip Respir Med 2019;14:17 doi: 10-1186/s40248-019-01792 eCollection 2019. 10.1186/s40248-019-0179-2 PMC6518652 31114679

[6] Dong X, Zhou J, Gou X, Li Y, Xu Y, Fu Q et al A retrospective analysis of distinguishing features of chest HRCT and clinical manifestation in primary Sjögren’s syndrome-related interstitial lung disease in a Chinese population. Clin Rheumatol 2018;37(11): 2981–2988. 10.1007/s10067-018-4289-6 30242640

[7] Wang Y, Hou Z, Qiu M, Ye Q. Risk factors for primary Sjögren’s syndrome-associated lung disease. J Thorac Dis 2018; 10(4): 2108–2117. 10.21037/jtd.2018.03.120 29850114 PMC5949459

[8] Flores-Chavez A, Kostov B, Solans R, Fraile G, Maure B, Feijoó-Massó C et al Severe life-threating phenotype of primary Sjögren’s syndrome: clinical characterization and outcomes of 1580 patients (GEAS-SS Registry). Clin Exp Rheumatol. 2018;36 Suppl 112(3):121–129. 30156546

[9] Flament T, Bigot A, Chaigne B, Henique H, Diot E, Marchand-Adam S. Pulmonary manifestations of Sjögren’s disease. Eur Resp Rev 2016;25 (140):110–123. 10.1183/16000617.0011-2016 PMC9487244 27246587

[10] Kampolis CF, Fragkioudaki S, Mavragani CP, Zormpala A, Samakovli A, Moutsopoulos HM. Prevalence and spectrum of symptomatic pulmonary involvement in primary Sjögren’s syndrome. Clin Exp Rheumatol 2018; 36 (Suppl 112) S91–101. 29846161

[11] Gao H, Zhang ZW, He J, Zhang J, An Y, Sun Y et al Prevalence, risk factors, and prognosis of interstitial lung disease in a large cohort of Chinese primary Sjögren’s syndrome patients: A case-control study. Medicine (Baltimore) 2018; 97(24):e11003. 29901591 10.1097/MD.0000000000011003 PMC6023797

[12] Sebastian A, Misterska-Skóra M, Silicki J, Sebastian M, Wiland P. Chest HRCT findings in patients with primary Sjögren’s syndrome. Adv Clin Exp Med 2017; 26(7):1101–1106. 10.17219/acem/68978 29211358

[13] Goh NS, Desai SR, Veeraraghavan S, Hansell DM, Copley SJ, Maher TM et al Interstitial lung disease in systemic sclerosis: a simple staging system. Am J Respir Crit Care Med 2008; 177(11):1248–54. 10.1164/rccm.200706-877OC 18369202

[14] Tauli B, Brauner MW, Mourey I, Lemouchi D, Grenier PA. Thin-section chest CT findings of primary Sjögren’s syndrome: correlation of pulmonary function. Eur Radiol 2002; 12:1504–1511. 10.1007/s00330-001-1236-7 12042961

[15] Herth FJF, Kirby M, Sieren J, Herth J, Schrim J, Wood S et al The modern art of reading computer tomography images of the lungs: quantitative CT. Respiration 2018; 95(1):8–17. 10.1159/000480435 28918422

[16] Ariani A, Silva M, Bravi E, Saracco M, Parisi S, de Gennaro F, et al Operator-independent quantitative chest computed tomography versus standard assessment of interstitial lung disease related to systemic sclerosis: a multi-center study. Mod Rheumatol 2015; 25(5):724–30. 10.3109/14397595.2015.1016200 25736361

[17] Sumikawa H, Jontok T, Yamamoto S, Yanagawa M, Inoue A, Honda O et al Computed tomography values calculation and volumen histogram analysis for various compute tomographic patterns of diffuse lung diseases. J Comput Assist Tomogr 2009; 33(5):731–738. 10.1097/RCT.0b013e31818da65c 19820502

[18] Shiboski CH, Shiboski SC, Seror R, Criswell LA, Labetoulle M, Lietman LM et al International Sjögren’s Syndrome criteria working group. 2016 American College of Rheumatology/ European League Against Rheumatism classification criteria for primary Sjögren’s syndrome: a consensus and data-driven methodology involving three international patient cohorts. Ann Rheum Dis 2017; 76(1):9–16. 10.1136/annrheumdis-2016-210571 27789466

[19] Von Elm E, Altman DG, Egger M, Pocock SJ, Gotzsche PC, Vandenbroucke JP et al The strengthening the Reporting of Observational Studies in epidemiology (STROBE) statement: guidelines for reporting observational studies. PLos Med 2007; 4(10): e296 10.1371/journal.pmed.0040296 17941714 PMC2020495

[20] Ash SY, Harmouche R, Vallejo DL, Villalba JA, Ostridge K, Gunville R et al Densitometric and local histogram-based analysis of computed tomography images of patients with idiopathic pulmonary fibrosis. Respir Res 2017; 18(1):45 10.1186/s12931-017-0527-8 28264721 PMC5340000

[21] Sambataro G, Ferro F, Orlandi M, Sambataro D, Torrisi SE, Quartuccio L, et al Clinical, morphological features and prognostic factors associated with Interstitial Lung Disease in primary Sjögren’s Syndrome: a systematic review from the Italian Society of Rheumatology. Autoimmun Rev, forthcoming 10.1016/j.autrev.2019.102447 31843713

[22] Ariani A, Silva M, Bravi E, Saracco M, Parisi S, de Gennaro F, et al Quantitative chest computed tomography is associate with two prediction models in interstitial lung disease related to systemic sclerosis. Rheumatology (Oxford) 2017; 56(6):922–927. 28160007 10.1093/rheumatology/kew480

[23] Moore OA, Goh NS, Corte T, Rouse H, Hennessy O, Thakkar V et al Extent of disease on high-resolution computed tomography lung is a predictor of decline and mortality in systemic sclerosis-related interstitial lung disease. Rheumatology 2013; 52(1):155–160. 10.1093/rheumatology/kes289 23065360

[24] Jacob J, Bartholmai BJ, Rajagopalan S, Brun AL, Egashira R, Karwoski R. Evaluation of computer-based computer tomography stratification against outcome models in connective tissue disease-related interstitial lung disease: a patient outcome study. BMC Med 2016; 14(1):190 10.1186/s12916-016-0739-7 27876024 PMC5120564

[25] Gao H, Zou YD, Zhang XW, He J, Zhang J, Sun Y et al Interstitial lung disease in non-sicca onset primary Sjögren’s syndrome a large-scale case-control study. Int J Rheum Dis 2018; 21(7):1423–1429. 10.1111/1756-185X.13334 29968329

[26] Chen MH, Chou HP, Lai CC, Chen YD, Chen MH, Lin HY et al Lung involvement in primary Sjögren’s syndrome: correlation between high-resolution computed tomography score and mortality. J Chin Med Asocc 2014; 77(2):75–82. 10.1016/j.jcma.2013.11.001 24342542

[27] Brito-Zerón P, Acar-Denizili N, Ng WF, Zeher M, Rasmussen A, Mandl T et al How immunological profile drives clinical phenotype of primary Sjögren’s syndrome at diagnosis: analysis of 10,500 patients (Sjögren Big Data Project). Clin Exp Rheumatol 2018; 36 (Suppl. 112): S102–S112. 30156539

[28] Fonseca VR, Romão VC, Agua-Doce A, Santos M, López-Presa D, Ferreira AC. The ratio of blood T follicular regulatory cells to follicular helper cells marks ectopic lymphoid structure formation while activated follicular helper T cells indicates disease activity in primary Sjögren’s syndrome. Arthritis Rheumatol 2018; 70(5):774–784. 10.1002/art.40424 29361207

[29] Reale M, D’Angelo C, Costantini E, Laus M, Moretti A, Croce A. MicroRNA in Sjögren’s syndrome: their potential role in pathogenesis and diagnosis. J Immunol Res 2018 6 7; 2018:7510174 10.1155/2018/7510174 eCollection 2018. 29977932 PMC6011049

[30] Guisado-Vasco P, de Luna Cardenal G, Martin-Garrido I, Luque-Pinilla JM, Fraile-Rodriguez G, Mateo JJ et al Assessment of interstitial lung disease in Sjögren’s syndrome by lung ultrasound: a pilot study of correlation with high-resolution chest tomography. Intern Emerg Med 2017; 12(3):327–331. 10.1007/s11739-016-1582-8 27900604

[31] Brito-Zerón P, Acar-Denzili N, Zeher M, Rasmussen A, Seror R, Theander E etl al. Influence of geolocation and ethnicity on the phenotypic expression of primary Sjögren’s syndrome at diagnosis in 8310 patients: a cross-sectional study from the Big Data Sjögren Project Consortium. Ann Rheum Dis 2017; 76(6): 1042–1050. 10.1136/annrheumdis-2016-209952 27899373

[32] Silva M, Milanese G, Seletti V, Ariani A, Sverzellati N. Pulmonary quantitative CT imaging in focal and diffuse disease: current research and clinical applications. Br J Radiol 2018; 91(1083):2017064 10.1259/brj.20170644 PMC5965469 29172671

[33] Judge PA, Lee JS, Ebstein E, Furukawa H, Dobrinskikh E, Gazal S et al MUC5B promoter variant and rheumatoid arthritis with interstitial lung disease. N Engl J Med 2018; 379(23):2209–2219. 10.1056/NEJMoa1801562 30345907 PMC6371965

[34] Chong DY, Kim HJ, Lo P, Young S, McNitt-Gray MF, Abtin F, et al Robustness driven feature selection in classification of fibrotic interstitial lung disease patterns in computed tomography using 3D texture feature. IEEE Trans Med Imaging 2016; 35:144–57. 10.1109/TMI.2015.2459064 26208309

[35] Thompson G, Mclean-Tooke A, Wrobel J, Lavender M, Lucas M. Sjögren’s syndrome with associated with lymphocytic interstitial pneumonia successfully treated with tacrolimus and abatacept as an alternative to rituximab. Chest 2018; 3; 153(3):e41–e43. 10.1016/j.chest.2017.12.010 29519308

[36] Yamano Y, Taniguchi H, Kondoh Y, Ando M, Kataoka K, Furukawa T et al Multidimensional improvement in connective tissue disease-associated interstitial lung disease: two courses of pulse dose methylprednisolone followed by low-dose prednisone and tacrolimus. Respiratology 2018; 23(11):1041–1048. 10.1111/resp.13365 30011421

